# More than just a color: Archaeological, analytical, and procedural aspects of Late Bronze Age purple-dye production at Cape Kolonna, Aegina

**DOI:** 10.1371/journal.pone.0304340

**Published:** 2024-06-12

**Authors:** Lydia Berger, Gerhard Forstenpointner, Peter Frühauf, Fabian Kanz

**Affiliations:** 1 Department of Classics, Classical and Early Aegean Archaeology, Paris Lodron University of Salzburg, Salzburg, Austria; 2 Institute of Morphology, University of Veterinary Medicine, Vienna, Austria; 3 Department of Analytical Chemistry, University of Vienna, Vienna, Austria; 4 Center for Forensic Medicine, Medical University of Vienna, Vienna, Austria; Austrian Academy of Sciences, AUSTRIA

## Abstract

Excavations in the Eastern Suburb of Bronze Age Aegina Kolonna revealed the destruction deposit of two sequenced Early Mycenaean buildings (phase Late Helladic IIA; 16th century BC). The older building is interpreted as a widely undisturbed production site of purple-dye based on indicative finds such as ceramic sherds containing analyzable quantities of pigment, high amounts of mollusk shells, and a few functional facilities. Chemical analysis by HPLC and malacological determination revealed that the banded dye-murex (*Hexaplex trunculus*) was used almost exclusively. The presence of crushing tools and a waste disposal pit provide insight into the technical process of dye production. Additionally, skeletal remains of heavily burnt infantile and juvenile piglets, kids, or lambs were found in the purple workshop area. The evidence may be better explained by ritual activities aimed at promoting the highly meaningful event of purple production, rather than by normal food consumption practices.

## Introduction

**Fig 1:**
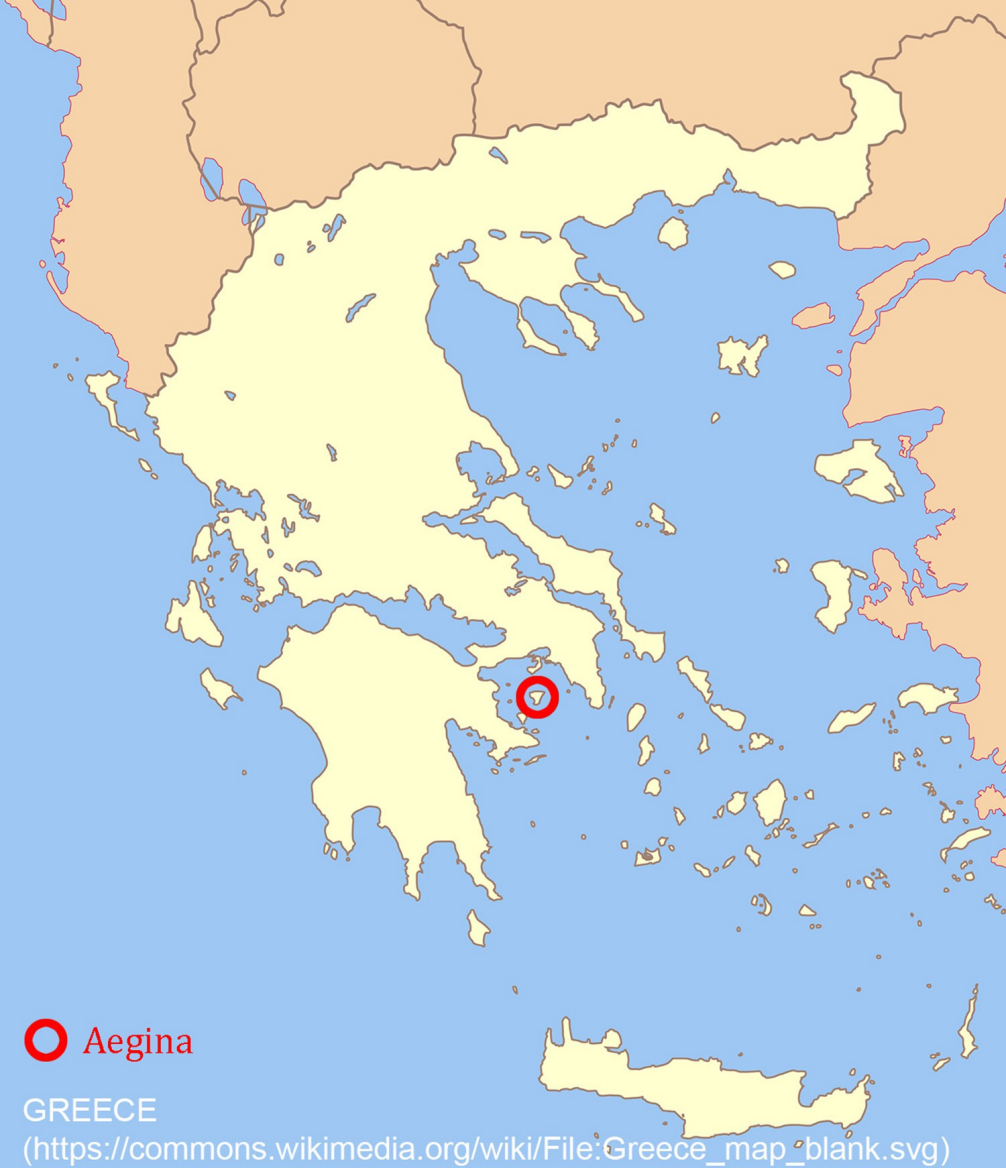
Location of the island Aegina. Map after https://commons.wikimedia.org/wiki/File:Greece_map_blank.svg.

**Fig 2:**
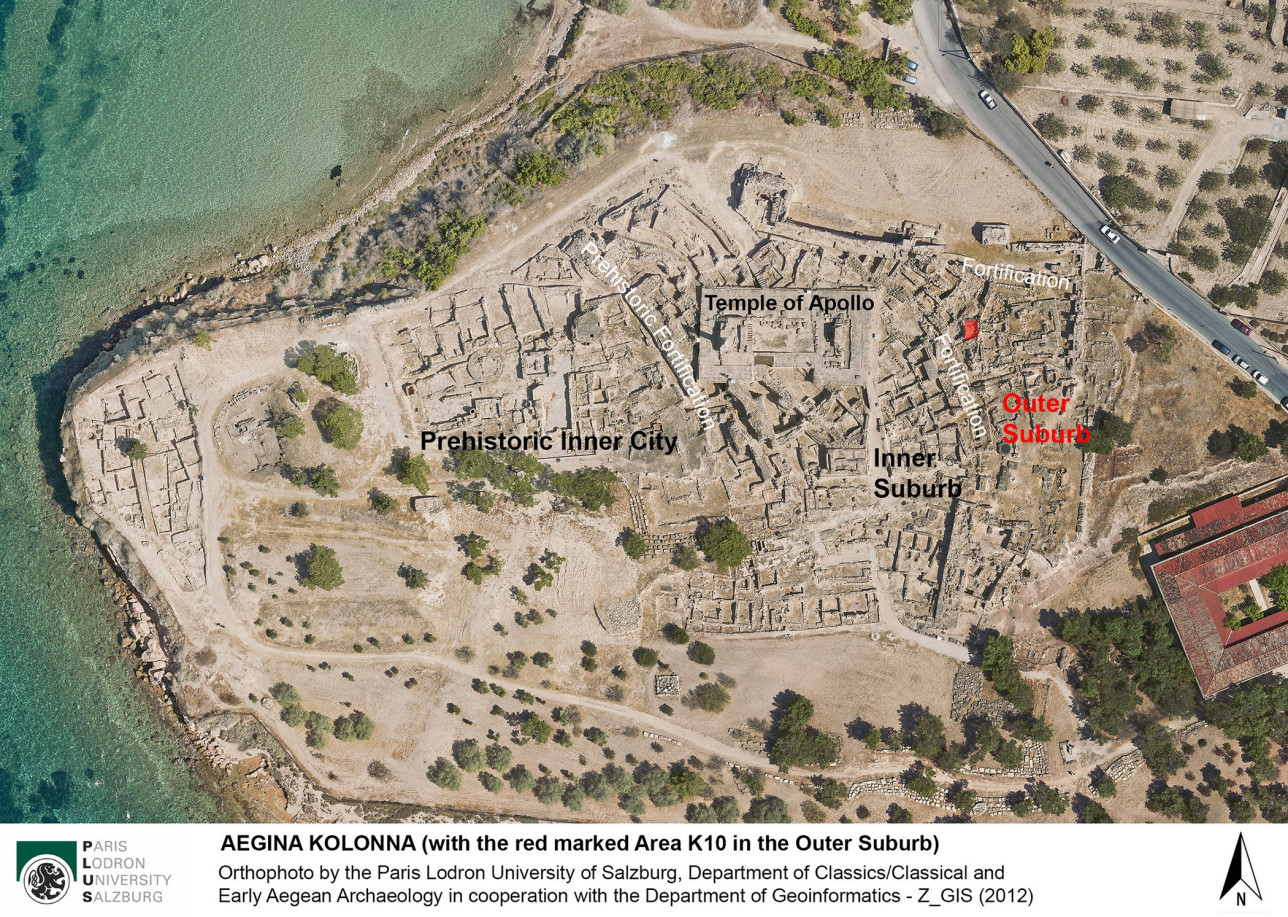
Orthophoto of Aegina Kolonna with Area K10 in the Bronze Age Outer Suburb marked in red.

The small island of Aegina in the center of the Saronic Gulf, between Attica, the Peloponnese, and the central Aegean Sea (Fig 1), has played a significant role in the cultural history of the Aegean for thousands of years. From Neolithic until Byzantine times (approximately 6th millennium BC– 10th century AD) the main settlement at Aegina was situated on a small, well-protected promontory on the north-western coast, called Cape Kolonna (Fig 2). In the 2nd millennium BC, this densely built and strongly fortified settlement experienced a period of economic prosperity and cultural heyday. Representative buildings, exceptional finds and rich graves indicate a complex economically stable social system integrated into an interregional trade network and the emerging cultures of the Middle and Late Bronze Age Aegean [1–3].

The striking Minoan influence, which is generally obvious in the material evidence of the Middle Bronze Age settlement at Kolonna [4–6], also suggests an adoption of purple production from Crete, where the technology had been known at least since the early 2nd millennium BC (Late MMI-MMII (1900–1700 BC)) [7–14].

The most frequent evidence for purple-dye production at prehistoric and historic sites comes from deposits of crushed marine snail shells [7, 9, 15, 16]. To obtain the desired dye, the hypobranchial glands of the snails, either extracted by opening the shell or as a component of the fully crushed mollusk body, were mixed with some salt water and left steeping for a few days in suitable containers, vats, or vessels [7, 15, 17, 18]. The choice of an adequate container allows the necessary control of the oxygen and light supply, which are important for obtaining the desired color shade. Although several taxa of the globally distributed gastropod family of *Muricidae* are suitable for the production of purple dye, only three Mediterranean species have been exploited at a relevant economic level. While the banded dye-murex (*Hexaplex trunculus*), the spiny dye-murex (*Bolinus brandaris*) and the red-mouthed rock shell (*Stramonita haemastoma*), are found throughout the Mediterranean, the latter is more common in the western part of the sea [19].

Pigment residues have rarely been preserved on the interior of containers/ceramic vessels, [20–23], on grinding or dyeing installations of workshops [24], on textiles [14, 25, 26] or as pigments of wall paintings [27, 28]. Chemical analysis of these pigments using High-Performance Liquid Chromatography (HPLC) revealed a significant difference between *Hexaplex trunculus* with rather high amounts of Monobromoindigotin (MBI) and lower contents of Dibromoindigotin (DBI) on the one hand, and *Bolinus brandaris* and *Stramonita haemastoma* with an opposite ratio of these substances on the other [29, 30].

At Kolonna, the exceptional finding of pottery fragments with remarkable quantities of well-preserved purple pigment, together with other archaeological evidence and significant zoological finds (for preliminary results see [31]), justifies the formulation of appropriate hypotheses, to be proven by detailed analysis:

- The Late Bronze Age settlement of Aegina Kolonna housed workshops for on-site production of purple-dye.
- Combined chemical and malacological analyses will facilitate the taxonomic evaluation of the purple-dye produced.
- Additional archaeological and bio-archaeological evidence will provide clues to the procedural peculiarities of purple-dye production at Bronze Age Aegina Kolonna.

## Archaeological evidence

In Aegina Kolonna, the technological knowledge of dye extraction from purple snails was first applied in the Middle Bronze Age period (1st half of the 2nd millennium BC) [11, 32, 33]. The occasional finds of mostly completely preserved purple snail shells from earlier contexts in Kolonna only prove the common collecting/fishing of the snails as food [11, 34]. Middle and Late Bronze Age evidence for purple-dye production, in form of large accumulations of crushed snail shells, has been found in various parts of the settlement [11, 33–36].

**Fig 3:**
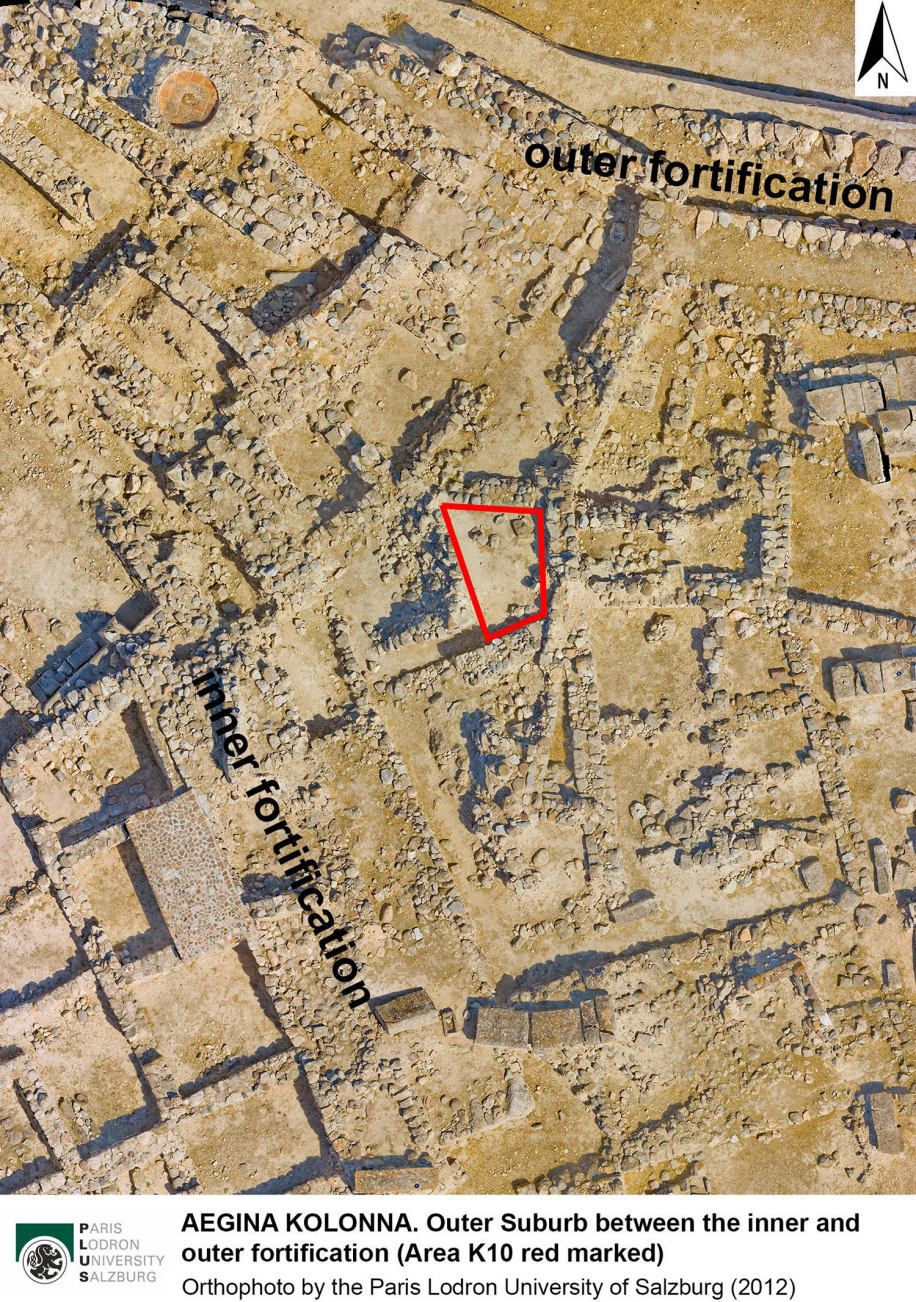
Orthophoto of the central part of the Outer Suburb in the northeast of Kolonna (Area K10 marked in red).

**Fig 4:**
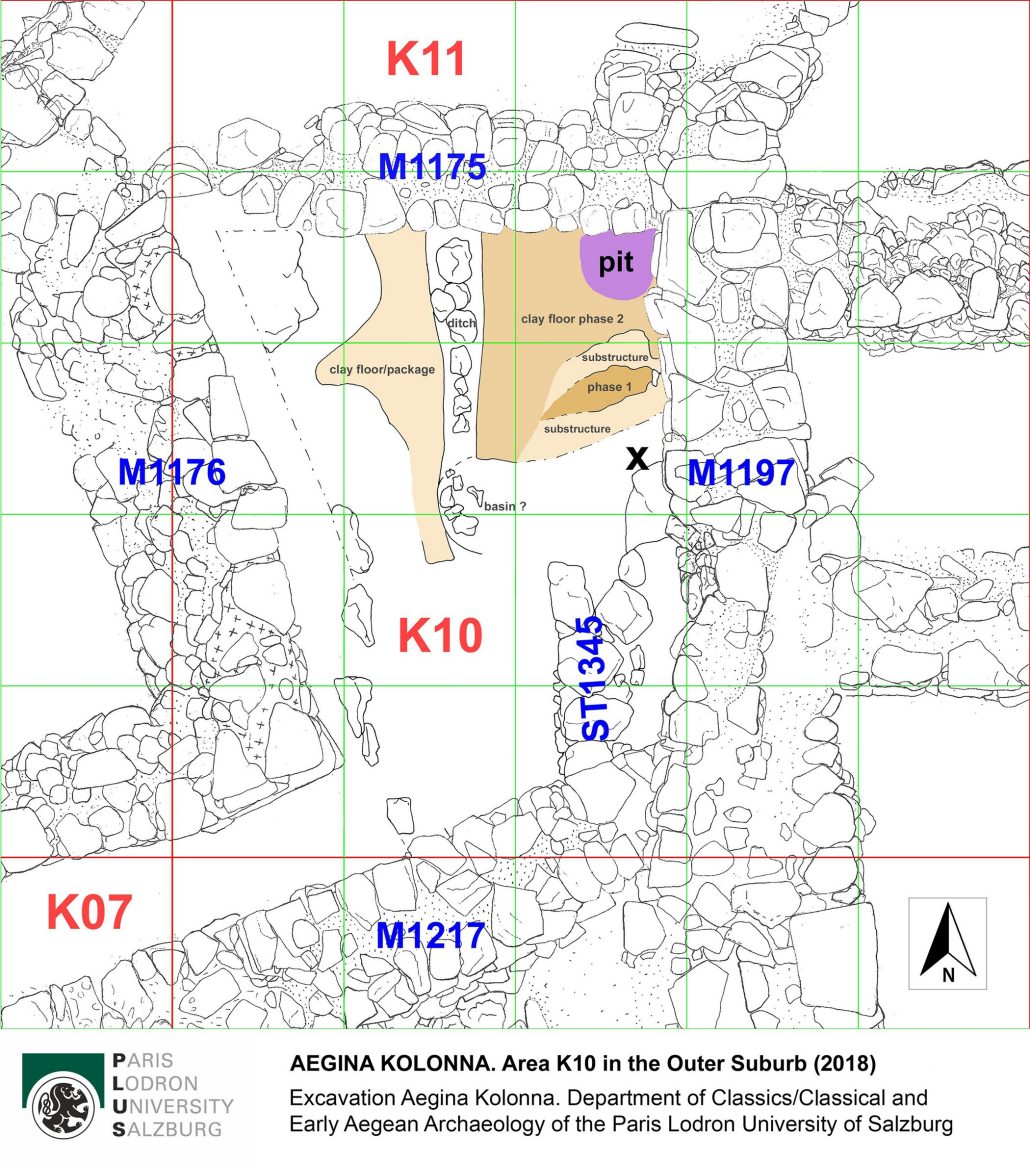
Plan of excavation Area K10 with the findspots of the ceramic fragments (= X) and the purple snail pit in the north-eastern corner.

Since 2015, archaeological investigations have focused on a small area in the Eastern Suburb of the prehistoric settlement, K10 (Figs 2–4), providing new results on the stratigraphy, dating and function of the suburbs for domestic and craft activities, particularly in the Late Bronze Age [37, 38].

Underneath a levelling layer the excavation in K10 revealed an approximately 0.75 m thick destruction deposit of two Early Mycenaean buildings (phase Late Helladic IIA (= LH IIA); 16^th^ century BC), which had collapsed one on top of the other. In the destruction debris of the older building, finds indicate a domestic context and are accompanied by evidence for various craft activities such as textile manufacture, stone tool production and small-scale grain processing [38]. Particularly noteworthy, however, is the clear evidence for on-site production of purple-dye at Kolonna.

In the central eastern part of Area K10, we found two ceramic fragments with adhering residues of purple pigment at the interior (see find spot “X” in Fig 4 and the ceramic fragments in Fig 5.1). The slightly water-soluble layer of paint emitted a fishy odor when exposed to water. The two mending sherds probably come from a closed pottery shape like a jug (cf. Fig 5.2) or a small jar. To prove their composition, the color pigments were chemically analyzed. The analyses and their results are discussed in more detail below.

**Fig 5:**
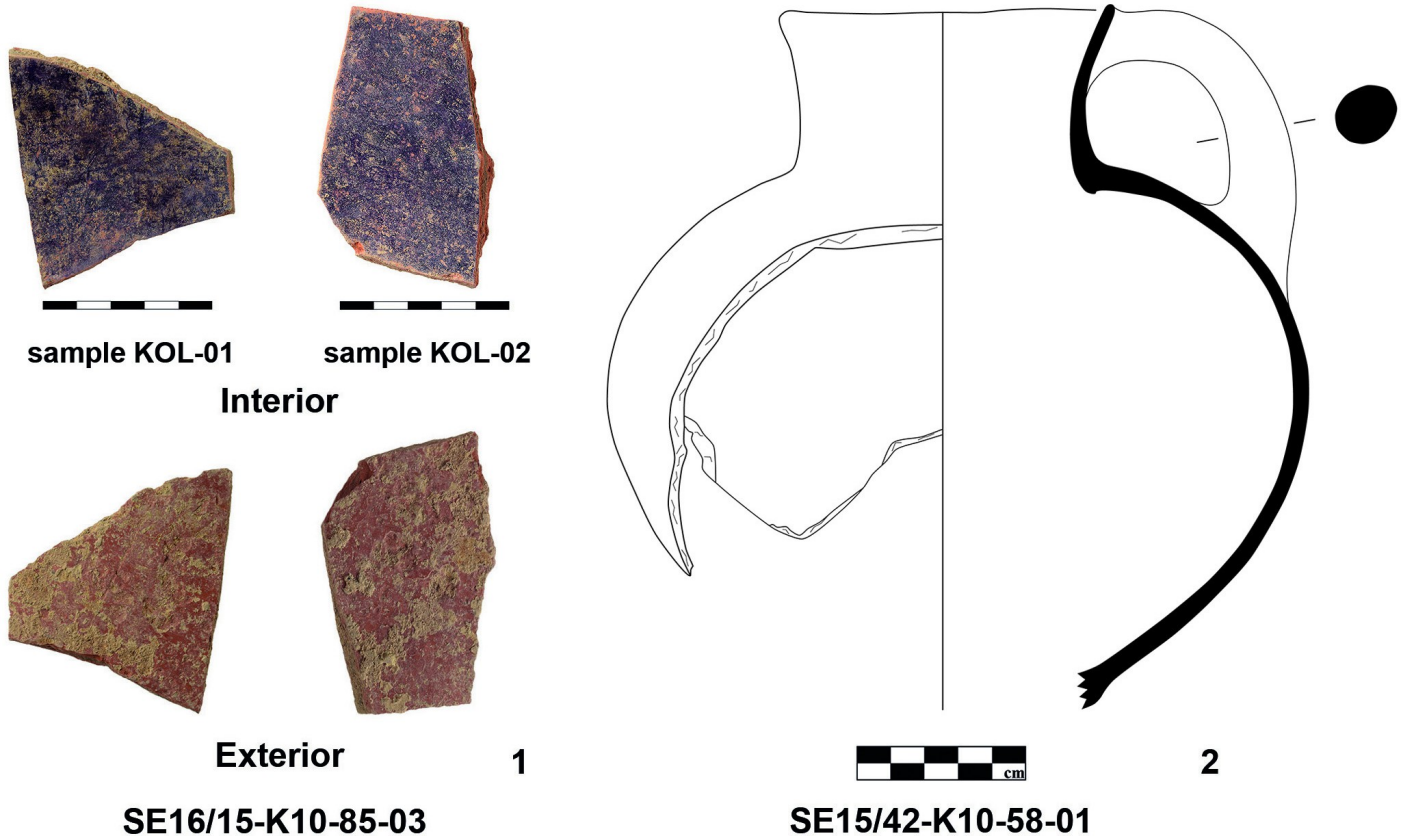
Pottery fragments from K10 with purple pigments at the interior (1), for comparison of shape another solidly painted jug from K10 (2). Photos and drawing by L. Berger.

So far, further six pottery fragments with small residues of purple pigment have been identified within the finds from K10. The fragments come from different pottery shapes, which could have been used either during the dye-processing or for storage or transport of the finished dye (cf. ceramic fragments with purple-dye residues: [9, 20, 23, 39]).

**Fig 6:**
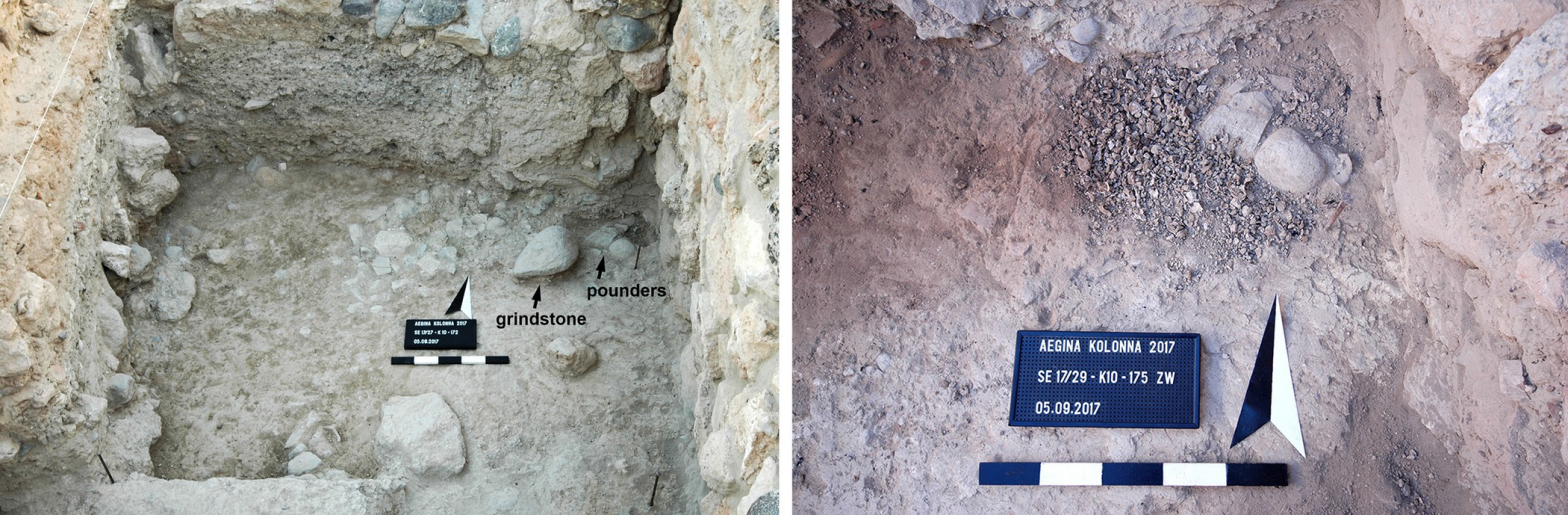
Purple snail pit in the north-eastern corner of Area K10: left before and right during excavation in 2017. Photos by L. Berger.

**Fig 7:**
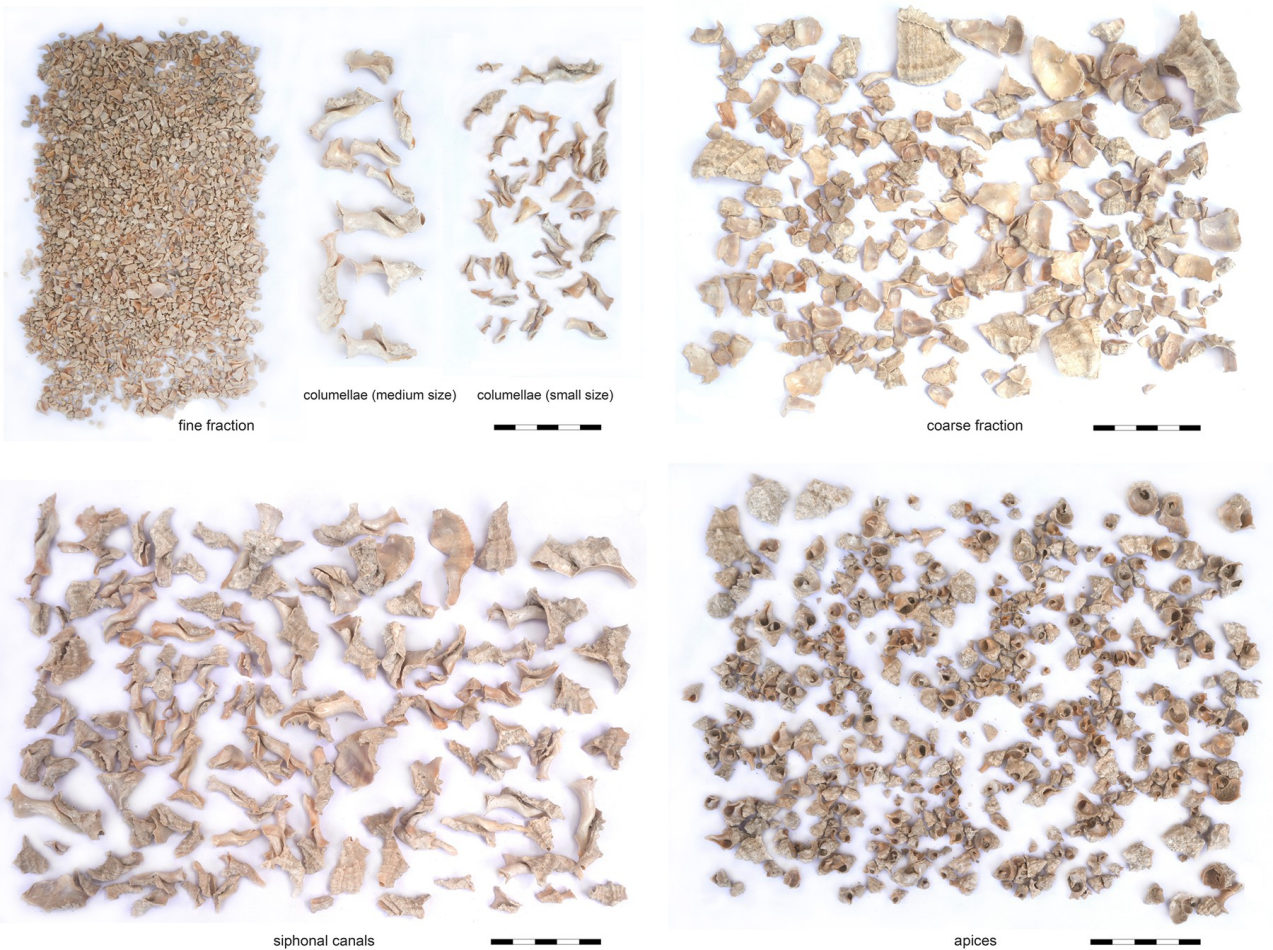
Selection of crushed purple snail shells from the pit in Area K10. Photos by L. Berger.

Additional evidence for purple-dye production in Late Bronze Age Kolonna is provided by a 0.5 m wide and 0.2 m deep pit in the north-eastern corner of K10 (Figs 4 and 6), filled with crushed purple snail shells (Fig 7) and buried by the destruction deposit. Above and within the shell grit we found three fist-sized stones, possibly used as pounders to crush the shells on a grindstone, which was placed aside (Fig 6) [9, 14]. The southern half of the pit was excavated and subjected to malacological analysis. The results of this study are detailed below.

Beneath the purple snail pit were the remains of a tamped clay floor. A narrow ditch, approximately 0.2 m deep and trapezoidal in cross-section, had been dug into the floor package only about 0.5 m from the pit (Fig 4). It ran in a north-south direction, sloping slightly to the north. The bottom of the ditch was either lined with a row of stones at the time of use or filled in with stones later. To the south was a shallow pit (basin?) filled with sediment, large fragments of pottery and smaller stones. The interpretation of the ditch as a drain/channel associated with a workshop or craft area is obvious. The connection with the production of purple-dye, as suggested by a comparison with a workshop area on the edge of the Middle Bronze Age settlement at Toumba/Thessaloniki [40] (cf. channel in [41]), cannot be proven at Kolonna. No murex fragments were found in the ditch. A contemporaneity with the purple snail pit discussed above can in any case be ruled out, as the most recent floor layer (phase 2 in Fig 4), above which the purple snail pit was located, covered the ditch.

## Analytical evidence for purple-dye and mollusk identification

### Sample preparation

**Fig 8:**
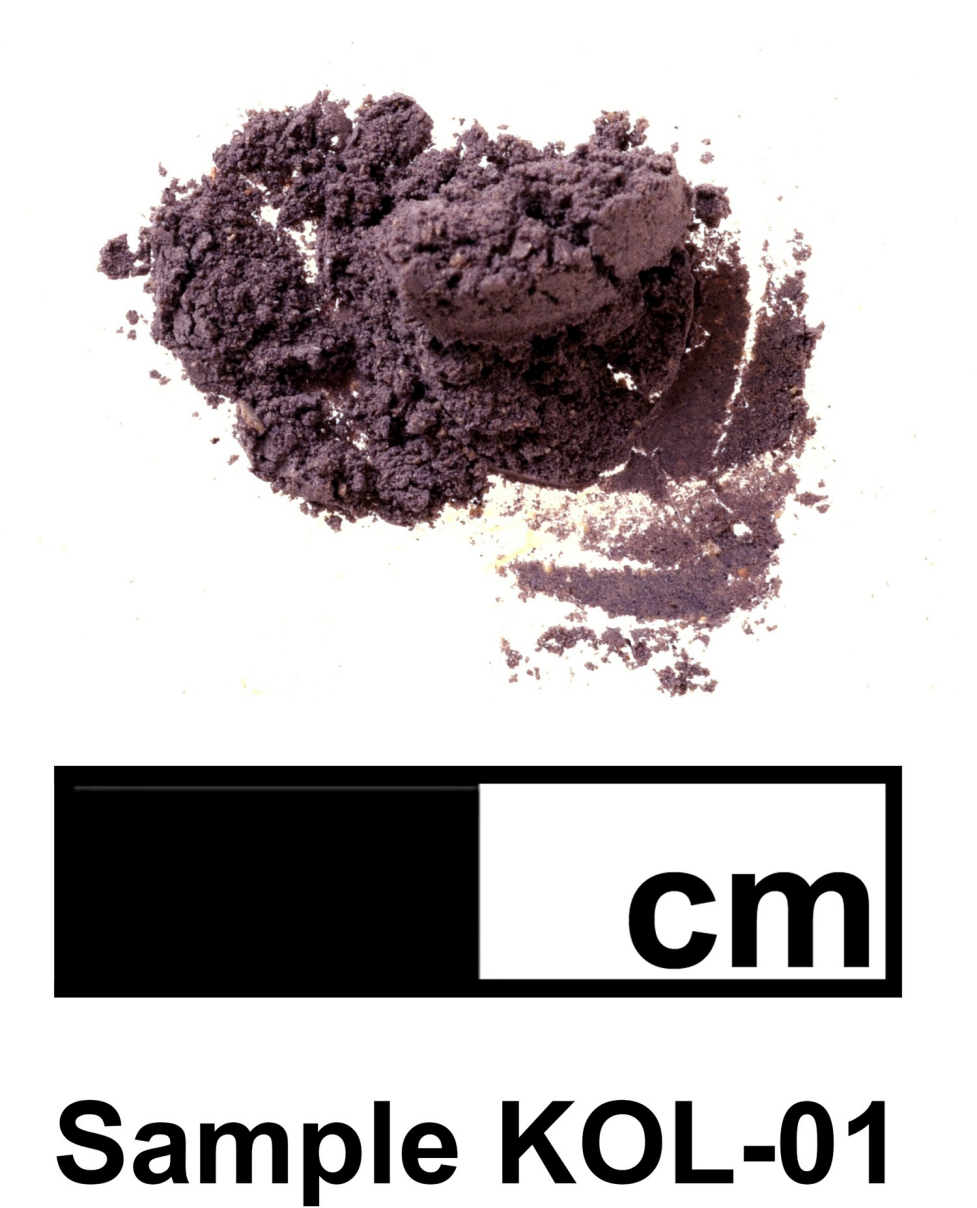
Purple pigment sample KOL-01 (from fragment SE16/15-K10-85-03). Photo by L. Berger.

Two recovered ceramic sherds with noticeable appositions (sample nos KOL-01 and KOL-02; Fig 5.1) were selected for HPLC analysis and sampled by scraping off the purple patina from the inner surface of the medium coarse vessel (Fig 8). Further sample preparation was carried out according to the protocol of Karapanagiotis et al. [42], using Dimethylsuloxide (DMSO) as the solvent of choice. 4.1mg for the sample KOL-01 and 3.9mg for the sample KOL-02 were each treated with DMSO at 80°C to extract the purple constituents, sonicated for 15 minutes and centrifuged at 13000rpm for 10 minutes. The supernatant was further aliquoted and analyzed by HPLC-UV (DAD).

### Color standards for comparison

Four compounds were selected as standards for the identification of colorants in HPLC. Indigotin (IND), indirubin (INR) and dibromoindigotin (DBI) were purchased from ABCR. 6’-bromoindirubin (6’-MBIR) was synthesized according to a published protocol [43]. The chemicals used for synthesis, 6-bromoisatin and 3-acetoxy-indole, were also obtained from ABCR. Reference standards of IND, INR, 6’-MBIR, DBI were weighed on an analytical balance (±0.1mg; Mettler Toledo) and diluted to 0.2mg/ml with DMSO (MERCK) to obtain stock solutions. To ensure good solvation, the standard solutions were heated to 80°C. Standard concentrations ranged from 0.2ng/μL to 50ng/μl for each substance. HPLC solvents and additives, acetonitrile and trifluoroacetic acid, were all obtained from Sigma-Aldrich, water was prepared in-house using a quartz bidestillation system.

### HPLC instrumentation

The HPLC system (Agilent Technologies) consisted of a 1200 binary pump, a 1200 autosampler, a 1200 column compartment, both thermostatically controlled and a 1200 diode array detector (DAD). All measurements were performed using a Phenomenex Gemini C18 column (3x150mm, 3μm particle size). TFA was added to obtain the best possible peak shape and to avoid unspecific binding to surface silanols. The method used was slightly modified from that previously published by Vasileiadou et al. [44] to improve the chromatographic performance of the HPLC system. By lowering the column temperature from 35°C to 15°C, good separation of the critical pair 6-MBIR and 6’-MBIR was achieved, giving a resolution of these two of 1.6. Five different wavelengths were chosen to monitor the chromatograms. For quantitative results, the chromatograms at 288nm were selected to compare the results obtained with previously published data. For calibration, a concentration range from 0.125ng/μl (0.25ng/μl for 6’-MBIR and DBI) to 5ng/μl was established as the working range. All four standards show good linearity with R^2^ > 0.999.

### Analysis results and discussion

In accordance with previously published data on the composition of Tyrian purple samples [42], it was decided to report the amount of the different coloring agents as their percentage in relation to the total content of determined species. Chromatograms at a wavelength of 288nm have been chosen to yield data comparable to others previously published [42, 45–49].

**Fig 9:**
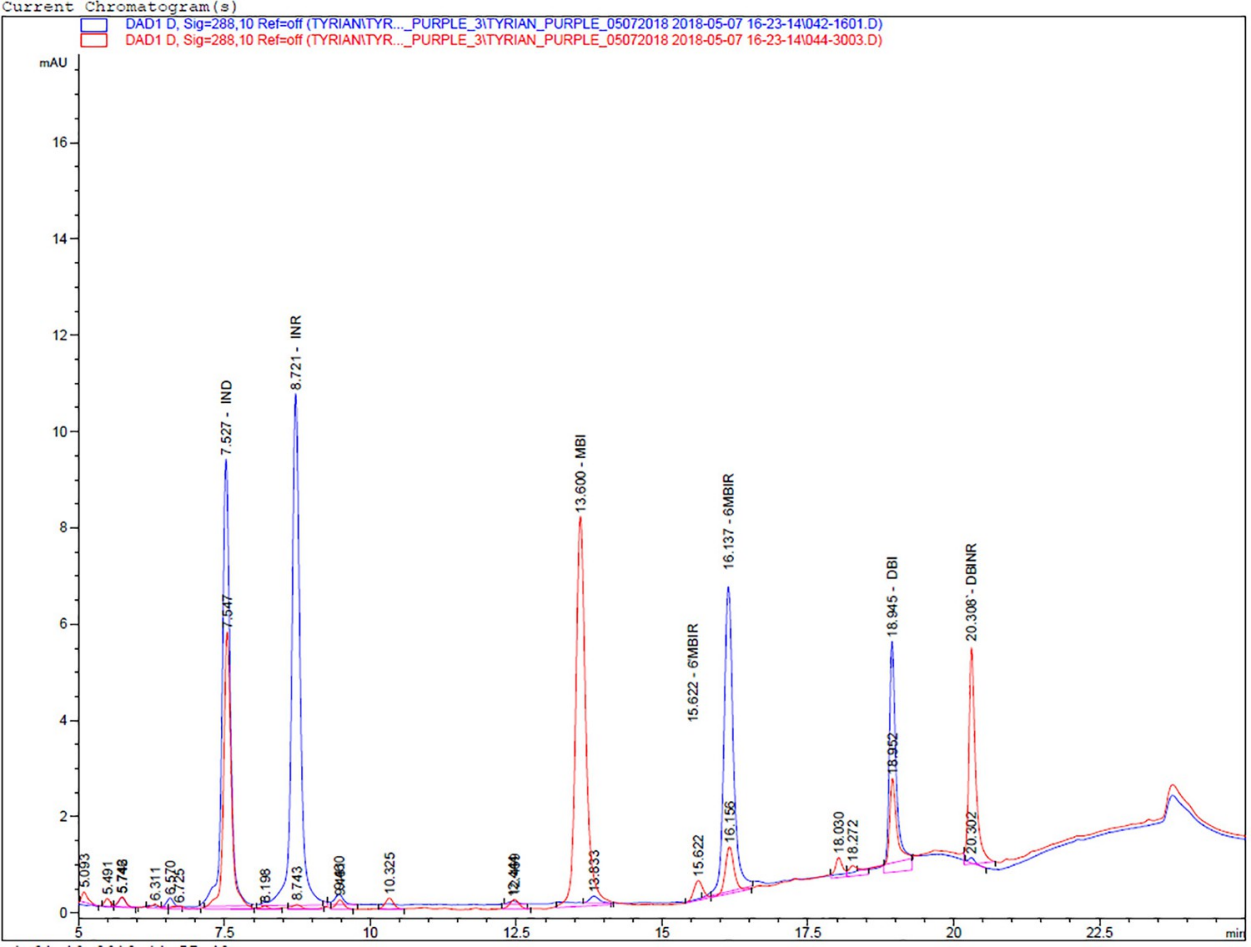
HPLC chromatograms at 288nm of the mixture of all color standards in comparison to the sample KOL-01.

Fig 9 shows the HPLC chromatograms at 288nm of the mixture of all color standards in comparison to the sample KOL-01. Percentage values for the Aegina samples KOL-01 and KOL-02, calculated from the integrated HPLC chromatograms are presented in detail in Table 1.

**Table 1:** Measured Tyrian purple components for the samples KOL-01 and KOL-02 as relative area (%) at 288nm.

		Percentage of integrated HPLC (288 nm) peak areas [%]							
ID	Origin	IND (blue)	INR (light purple)	MBI (purple)	6‘MBIR (red)	6MBIR (light red)	DBI (dark red)	DBIR (red)	Other
KOL-01	Aegina	23,9	0	44,5	2	4,7	6,3	14,9	3,7
KOL-22	Aegina	21,8	0	39,1	1,9	2,7	11,8	15,1	7,6

The results reported in Table 1 for the sampled appositions on the two ceramic sherds (KOL-01 and KOL-02) are qualitatively and quantitatively similar, showing moderate amounts of IND, high amounts of MBI, and moderate amounts of DBI and DBIR. INR was not detected and monobromoindirubins (6’MBIR and 6MBIR) were detected in very small amounts.

Some coloring compounds of true purple, such as indigotin (IND) and its isomer indirubin (INR), may also originate from indigoid dyes of plant origin, such as those from *Indigofera spp*. and *Isatis tinctoria*. A strong identifying marker compound of Tyrian purple is 6,6′-dibromoindigotin (DBI) [50], which was identifiable in both samples (KOL-01 and KOL-02) in significant amounts, 6.3 and11.8% respectively.

Relatively high proportions of indigotin (23.9 and 21.8%) in the samples suggest that the dye originated from the banded dye murex, *Hexaplex trunculus*, rather than *Bolinus brandaris* and *Stramonita haemastoma*, as dyes from these two gastropod typically have very low, close to zero, proportions of IND [49].

**Fig 10:**
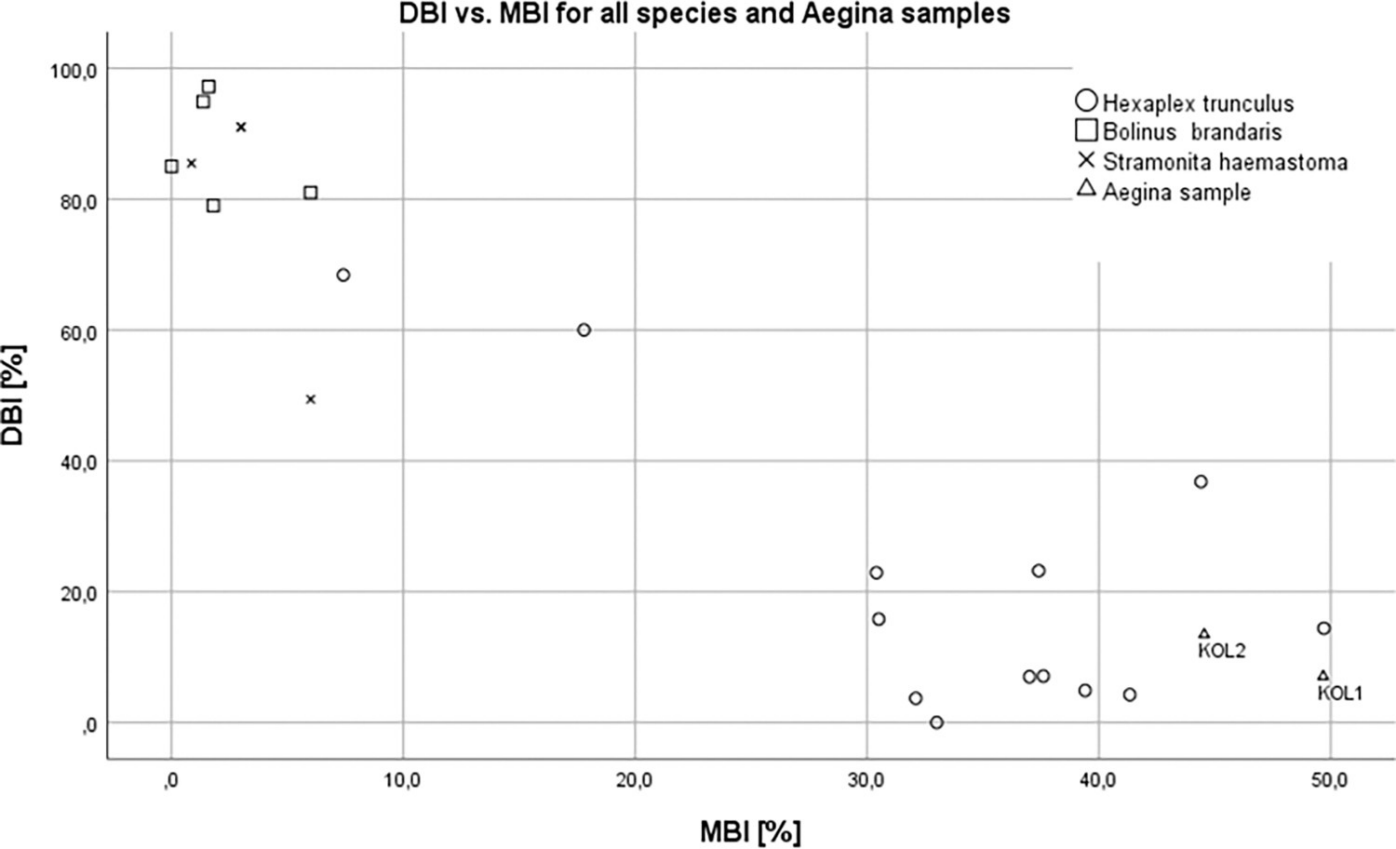
DBI vs. MBI data for all three mollusks species and Aegina samples KOL-01 and KOL-02.

To further support this indication of origin, we decided to plot previously published MBI and DBI data for all three mollusks species [42, 45–49] and compare them with the data for the two Aegina samples (Fig 10).

The comparison of the data in Fig 10 shows a strengthened evidence for the possible origin of the Aegina samples from *Hexaplex trunculus* by the low DBI values (6.3 and 11.8%) and especially by the very high percentages of MBI (44.5 respectively 39.1%). Such high MBI values are so far only known for pigments obtained from *Hexaplex trunculus* samples.

### Analytical conclusion

In accordance with previous evidence [26, 29] our results again demonstrate that chemical analysis of preserved purple pigment remains allows the identification and rough quantification of exploited muricid taxa. In the case of Late Bronze Age Aegina, it is very likely that almost exclusively *Hexaplex trunculus* specimens were processed.

## Faunal remains from K10

In this paper we discuss finds from the destruction layers of the two LH IIA Early Mycenaean buildings in the Area K10, as well as the composition and taphonomy of the ‘purple snail pit’, which is assigned to the destruction deposit of the older LH IIA building. Although the analysis of the vertebrate and mollusk remains from the lowermost debris layers of the earlier building is not yet complete, preliminary screening of the small number of remaining samples does not indicate significant divergences from the overlying evidence.

Consistent dry sieving of the excavated soil (1cm mesh) and wet sieving of the material from the pit yielded large quantities of small splinters, but bones and mollusk shells were found to be in excellent condition. Determination was carried out on-site using a mobile osteological reference collection that is housed at the Austrian Archaeological Institute in Athens. While all diagnostic procedures followed the established standards of archeozoological research, the estimation of culling ages was mainly based on Habermehl, Payne, and Hongo [51–53], the differentiation of sheep and goats among others to Boessneck et al. as well as Zeder and Lapham [54, 55].

### Vertebrate remains

The excavation yielded a total of 2592 mammal remains, of which 1455 could be identified as determinable up to genus level (Table 2). Even though there is no significant predominance of skeletal elements, these finds most likely represent alimentary waste belonging to consumers that had been involved in the production of purple dye. Taxonomically, a large proportion of the assemblage represents ovicaprines (54%), of which the determinable part branches to 69% sheep and 31% goats. While the proportion of bovine remains (9%) is much lower than in other Late Bronze Age samples [56, 57], the proportion of pig bones (37%) is remarkably high. Mammal game remains are present in very small quantities. Red deer is represented by one antler fragment and one calcaneus, hare by one thigh bone and wild boar by one rib, unmistakable by its size and form. A large horncore from an aurochs could be rather interpreted as a prestigious object rather than as actual hunting prey, and a tibia from a hedgehog shows cut marks, suggesting unexpected culinary practices. Six remains of columbiform birds, mainly turtle doves (*Streptopelia turtur* Linné, 1758) and one seagull (*Larus sp*. Linné, 1758), are very reminiscent of similar finds from Middle Bronze Age Aegina [58] and Middle Minoan Kommos [59]. Analysis of fish bones, which are present in small quantities, has not yet been completed.

**Table 2:** Mammal remains from K10 –overview on determined species and body parts.

	B	Bp	O-C	O	C	S	Cn	Ee	Eu	m	l	total
Head	12	1	46	17	8	78						**162**
Trunk	22	0	94	4	1	46	0	0	1			**168**
Legs	10	0	65	5	3	43	1	1				**128**
Feet	0	0	18	4	2	15	1	0				**40**
Toes	3	0	2	1	0	6						**12**
long bones										164	11	**175**
cortical bone										97	30	**127**
cancellous bone										11	4	**15**
**Total**	**47**	**1**	**225**	**31**	**14**	**188**	**2**	**1**	**1**	**272**	**45**	**827**
NISP %	9,2	0,2	44,1	6,1	2,7	36,9	0,4	0,2	0,2			

Abbr.: B–cattle, Bp–aurochs, O-C–sheep/goat, O–sheep, C–goat, S–domestic pig, Cn–dog, Ee–horse, Eu–equine (hybrid), m–medium body size, l–large body size.

### Burnt vertebrate remains

**Fig 11:**
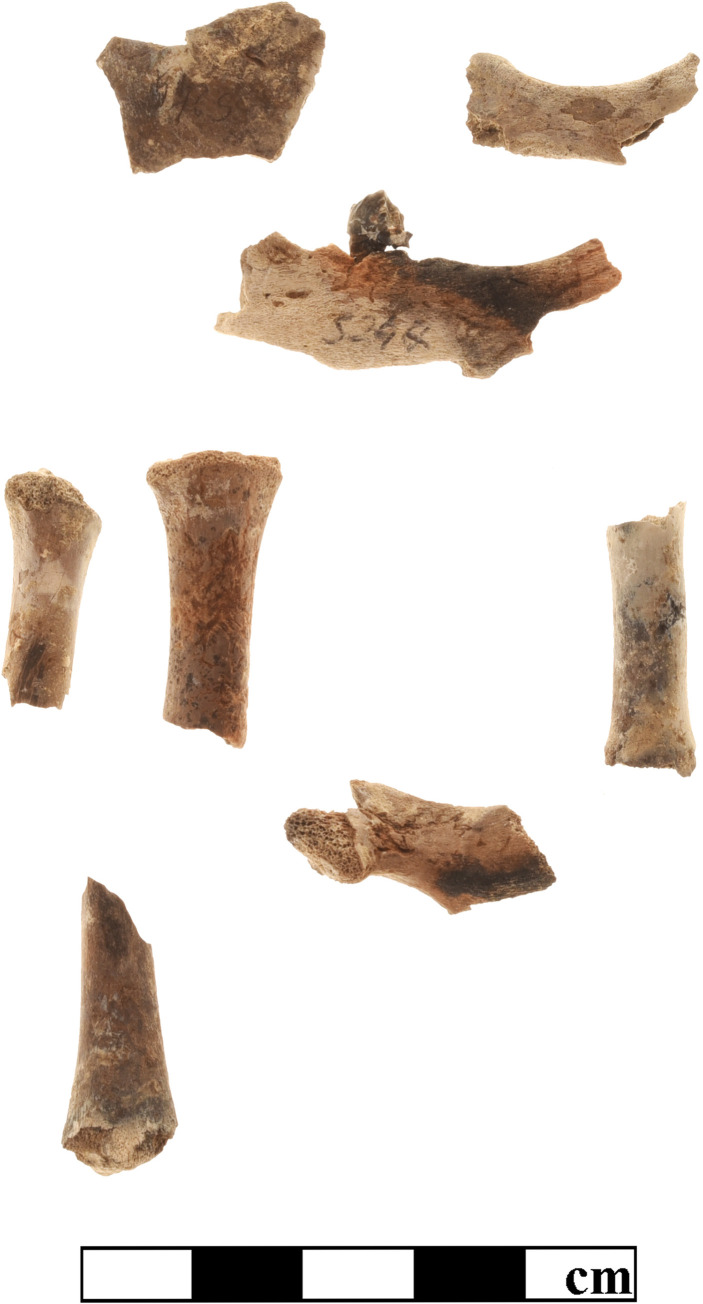
Remains of at least two piglets at the age of 1–2 months from SE17/04-K10. Photo by G. Forstenpointner.

**Fig 12:**
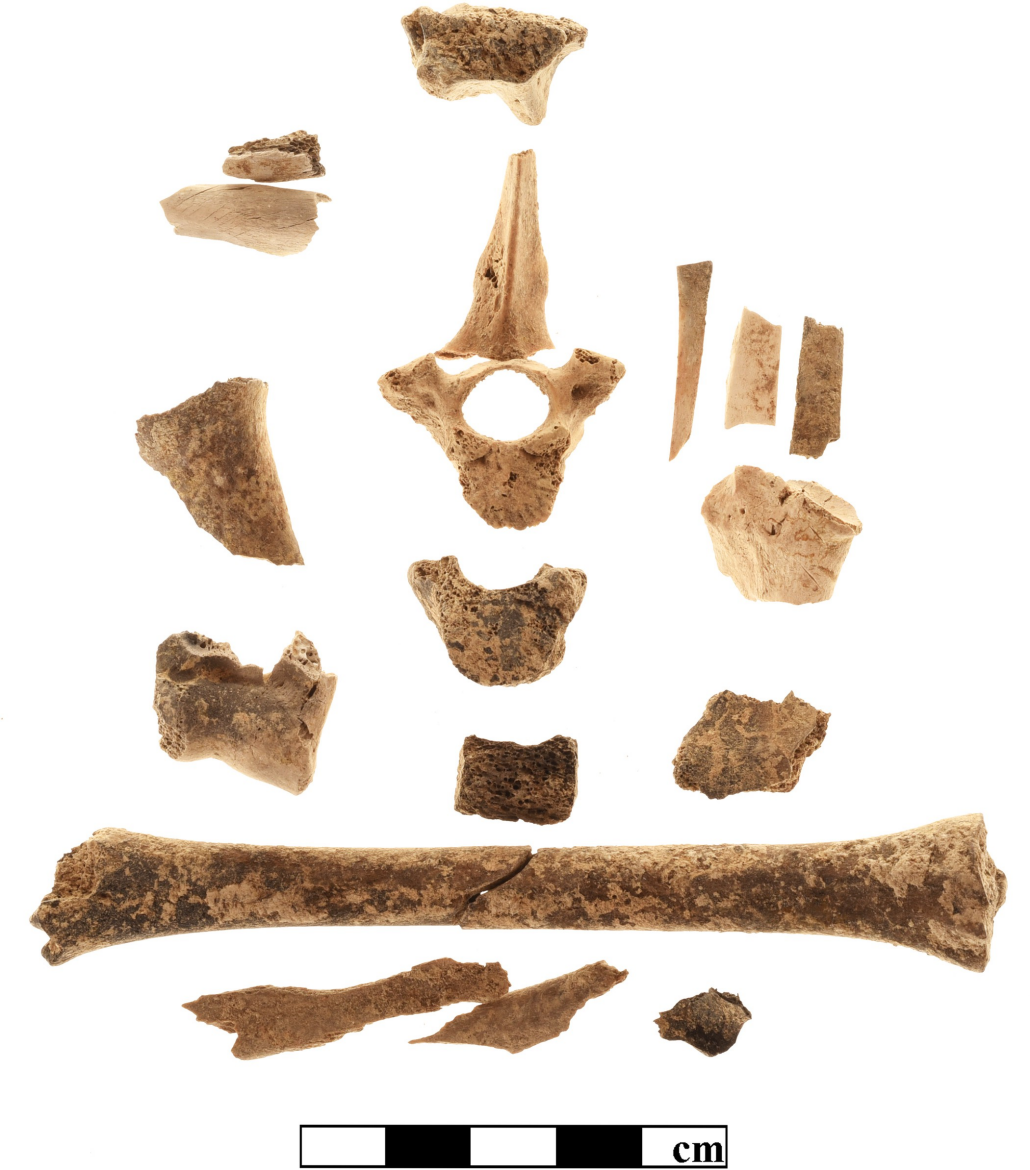
Remains of maybe one lamb or kid from SE17/25-K10. Photo by G. Forstenpointner.

Although patterns of mammalian exploitation are not the primary objective of the present study, one particular finding should be mentioned in relation to the process of purple production. The total mammal bone assemblage from K10 comprised 4,8% of burnt fragments, restricted to ovicaprines, sus, and bos. Approximately three quarters of these finds appeared calcined, with a grey to white color and brittle texture, indicating a relatively high temperature of 500–700° C. The remaining fragments appeared blackish charred, due to heat exposure of up to 500° C [60]. With respect to the remains of immature individuals, in particular of infantile (neonate– 4 months), rather than juvenile (5–12 months) ovicaprines and pigs, we recorded a completely different percentage of burnt specimens. Table 3 shows strikingly high proportions of calcined bones from young piglets and lambs or kids, but these finds are not homogeneously distributed throughout the sample, being mainly restricted to a few clusters of stratigraphic units from the destruction layers of both the earlier and later Early Mycenaean buildings (phase LH IIA), always associated with more or less large ash deposits and charcoal. Remarkably, the majority of the finds form distinct deposits, Figs 11 and 12 show the remains of at least two piglets of 1–2 months of age from SE17/04 and of maybe one lamb or kid from SE17/25. Based on the skeletal representation, it is likely that the burning of these very young domestic animals was holocaustic, which is not a common feature of consumption. The function of this procedure should be discussed in detail.

**Table 3:** Burnt bones from K10: An overview with respect to culling stages.

	O-C	S	B	other	Indet	total
**Total**	**784**	**496**	**158**	**13**	**1141**	**2592**
Calcined	30 (3,8%)	30 (6%)	0	0	37 (3,2%)	**97 (3,7%)**
Charred	13 (1,7%)	2 (0,3%)	2 (1,3%)	0	11 (1%)	**28 (1,1%)**
Total Infantile	70	64	1	0		**135**
Calcined	14 (20%)	23 (36%)	0	0		**37 (27,4%)**
Charred	9 (12,9%)	1 (1,7%)	0	0		**10 (7,4%)**
Total Juvenile	35	64	5	0		**104**
Calcined	1 (2,9%)	2 (3,1%)	0	0		**3 (2,9%)**
Charred	0	1 (1,7%)	0	0		**1 (1%)**

Abbr.: O-C–sheep/goat, S–pig, B–cattle

### Invertebrate remains–gastropods

Due to different recovery methods, we distinguish two main samples of invertebrate remains: Dry-sieved specimens from the destruction layers of the two superimposed Early Mycenaean buildings and the wet-sieved content of the “snail-pit”. Terrestrial gastropods, which occurred in considerable numbers in the dry-sieved samples, are not the aim of this study and won’t be discussed further.

**Fig 13:**
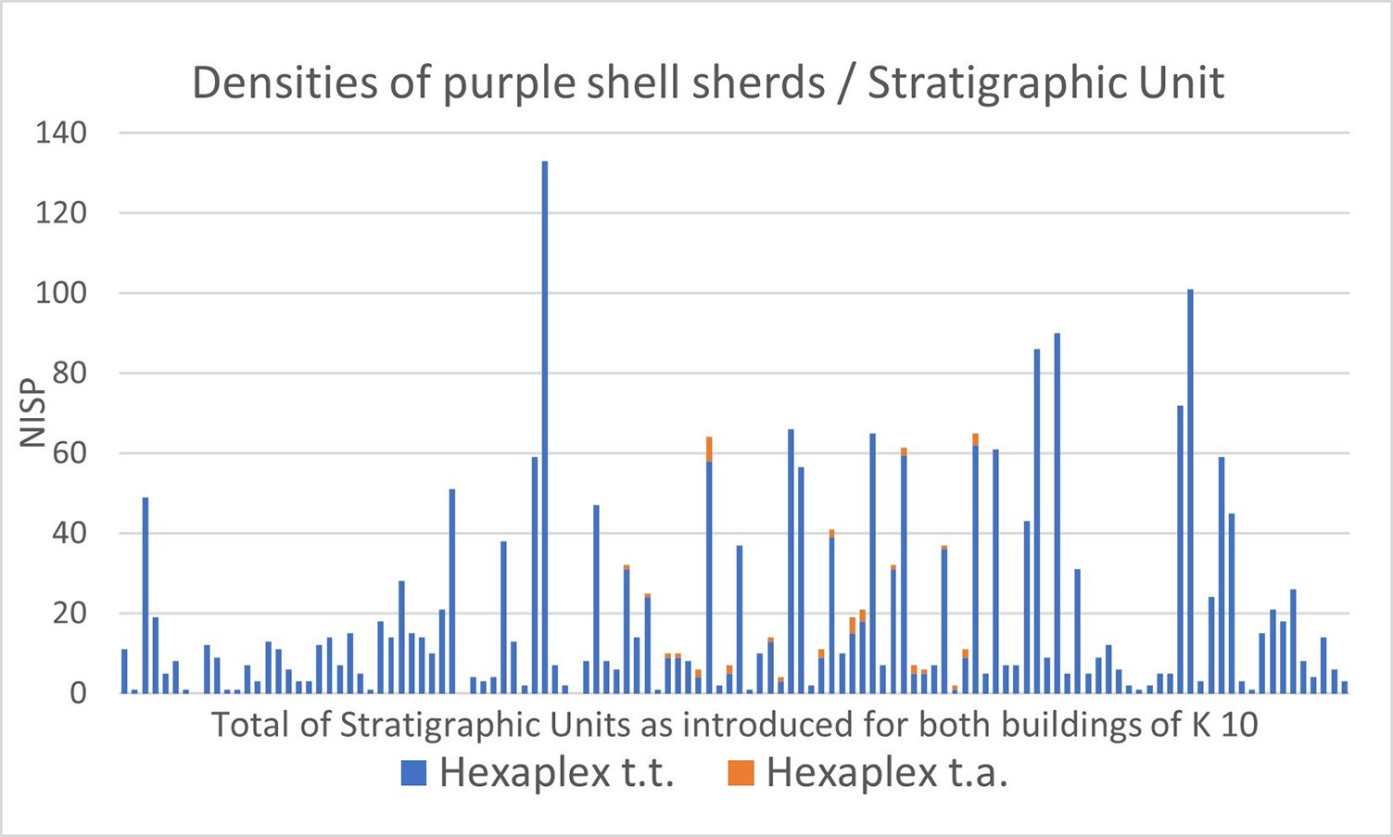
Densities of purple shell sherds per stratigraphic unit. Abbr.: Hexaplex t.t.–Hexaplex trunculus trunculus, Hexaplex t.a.–Hexaplex trunculus armigerus.

**Fig 14:**
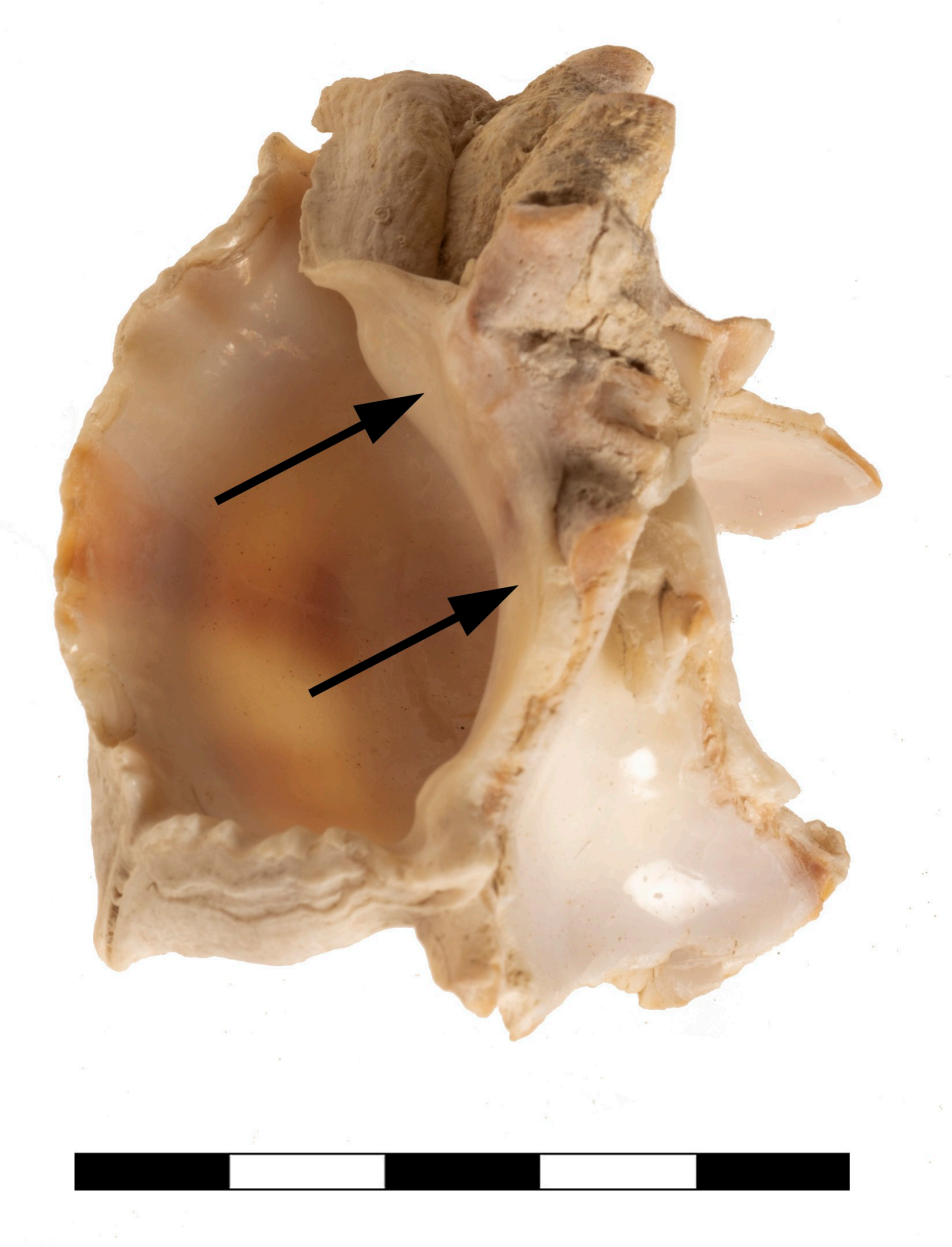
Shell fragment with rounded edges from K10. Photo by G. Forstenpointner.

Accompanied by a wide variety of marine gastropod taxa (Table 4), the composition of the dry-sieved samples is largely dominated by crushed shells of the banded dye-murex, *Hexaplex Trunculus* (Linné, 1758), present in almost all stratigraphic units, albeit in variable densities (Fig 13). The total number of counted *Hexaplex* shell fragments represents 84,3% (n = 2339) of the marine gastropod sample, the number of siphonal canals amounts to an MNI of 674, representing 73,6% of the total MNIs. A very small subgroup (NISP = 42, MNI = 30) of *Hexaplex* specimens represents the conspicuous variety of *Hexaplex trunculus f*. *armigerus* (Settepassi, 1970), which in Late Roman times indicated luxurious dishes of seafood, obviously due to its huge dimensions and long, curved spines and perhaps also to its rare availability [61]. *Bolinus brandaris* (Linné, 1758), the spiny dye-murex (MNI = 11) and *Stramonita haemastoma* (Linné, 1767), the red-mouthed rock shell (MNI = 4), as the two other relevant and doubtlessly available Mediterranean purple snails, occur in extremely low numbers. The shells of the latter and of all other taxa appear more or less preserved and most probably represent alimentary waste. In particular, *Patella caerulea* and *rustica* (both Linné, 1758, MNI = 151), as well as several species of *Phorcus* (Risso, 1826, MNI = 48) and *Cerithium vulgatum* (Bruguière, 1792, MNI = 18) might have been eaten, while occasionally occurring species could be interpreted as accidental additions to the snail catch. About 20% of the shell fragments have rounded edges, most likely caused by trampling attrition of the floor-embedded sherds (Fig 14).

**Table 4:** Marine gastropods from K10: Dry-sieved samples.

CAENOGASTROPODA	NISP	MNI
**Muricoida**		
*Hexaplex trunculus* (Linné, 1758)	2297	644
*Hexaplex tr*. *F*. *armigerus* (Settepassi, 1970)	42	30
*Bolinus brandaris* (Linné, 1758)	19	11
*Stramonita haemastoma* (Linné, 1767)	4	4
*Ocinebrina aciculata* (Lamarck, 1822)	1	1
*Muricidae* (Rafinesque, 1815)	1	1
**Cerithoidea**		
*Cerithium vulgatum* (Bruguière, 1792)	47	18
**Buccinoidea**		
*Euthria cornea* (Linné, 1758)	16	9
*Aptyxis syracusana* (Linné, 1758)	2	2
*Pisania striata* (Gmelin, 1791)	1	1
**Tonnoidea**		
*Tonna galea* (Linné, 1758)	4	2
*Charonia sp*. (Gistel, 1847)	2	2
*Galeodea echinophora* (Linné, 1758)	1	1
**VETIGASTROPODA**		
**Trochoidea**		
*Phorcus turbinatus* (Born, 1780)	67	28
*Phorcus articulatus (Lamarck*, *1822)*	3	3
*Phorcus mutabilis* (Philippi, 1846)	1	1
*Phorcus sp*. (Risso, 1826)	16	12
*Steromphala umbilicalis* (da Costa, 1778)	3	3
*Steromphala divaricata* (Linné, 1758)	1	1
**Fissurelloidea**		
*Diodora graeca* (Linné, 1758)	2	2
**Patellogastropoda**		
*Patella caerulea* (Linné, 1758)	178	108
*Patella rustica* (Linné, 1758)	56	40
*Patella sp*. (Linné, 1758)	10	3
**total**	**2774**	**916**

Abbr.: NISP–Number of Identified Specimens, MNI–Minimum Number of Individuals

The wet-sieved sample from the “snail-pit” comprises only a quarter of the entire deposit, as the analysis took place on-site and we had to consider the limited time frame of our research campaign. With 4119 counted faunal remains, including 4058 (98,5%) shell fragments of *Hexaplex trunculus* (Linné, 1758), a main feature of the sample is its remarkably high degree of homogeneity. This characteristic may provide an indication of the methods used to catch purple shellfish: experimental attempts [15] have shown that baited traps also attract other carnivorous gastropod species, in particular *Buccinulum corneum*, which form a part of the catch and finally appear in the sample of shell remains. In comparison, hand harvesting of purple shells is likely to produce more homogeneous catches than other methods [62]. The degree of fragmentation is very high, undoubtedly due to the recovery of finds by wet-sieving, and indicates deliberate pounding of the shells, not just punctual crushing to remove the hypobranchial gland. Similar evidence has been reported from Cypro-Classic Paphos [63]. All fragments appear freshly broken, which may support a procedural interpretation of periodic emptying and refilling of the waste pit during the process of purple-dye production. Counting the number of siphonal canals, the analyzed sample represents an MNI of 490, which means that one filling of the entire “snail-pit” amounts to 2000 individuals. Although there is no evidence to indicate the frequency of emptying and refilling of the pit, the trampling traces on the shell fragments from the surrounding floors (Fig 14), as well as the presence of these finds in all analyzed layers (Fig 13), point to continuous, most likely professional, activities of dye-production in Area K10.

The sample from the pit includes all anatomical parts of the snail shell in reasonable quantities (Table 5). Assessment of individual shell sizes by categorizing sufficiently well-preserved apertures and siphonal canals (Table 5) revealed a very high percentage of small individuals. This finding offers at least three interpretative approaches: again, the applied fishing method could be a crucial factor in the composition of the catch, as baited traps attract juvenile individuals no less than adults [15]. Alternatively, one could consider Aristotle’s remarks on purple snails (HA V, 547a 6–9), which may indicate a certain selectivity in the catching of these mollusks due to the dependence of the color hue on size of the animal [62]. Finally, given that the small individuals represent juvenile stages, also an overexploitation of the local snail population appears imaginable. Similar evidence has been reported for various species from different sites and ages [64, 65], including a Byzantine purple production site [66]. However, recent studies have shown that a proper understanding of the interaction between fishing pressure and population density in muricid gastropods requires further ambitious historical ecology studies [67].

**Table 5:** Anatomical parts and size classes of Hexaplex trunculus shells from the “snail pit” in K10.

	Total	assessable shell fragments		
		l	m	s
Apex	419			
Body whorl	1315			
Aperture	144	2	25	98
Columella	338	1	22	78
Siphonal canal	490	3	7	64
indefinable	1352			
total	4058			

Abbr.: l–large (width 35–50), m–medium (width 20–35), small (width <20)

For the sake of completeness, the “snail-pit” sample also comprised four shells of *Tritia reticulata* (Linné, 1758), one of *Patella caerulea* (Linné, 1758), three of the terrestrial gastropod family *Zonitidae* (Moerch, 1864), two mantle fragments of the bivalve *Pinna nobilis* (Linné, 1758) and 50 specimens (16 shell fragments and 34 spines) of the purple sea urchin (*Paracentrotus lividus* Lamarck, 1816).

### Invertebrate remains–bivalves and other taxa

**Fig 15:**
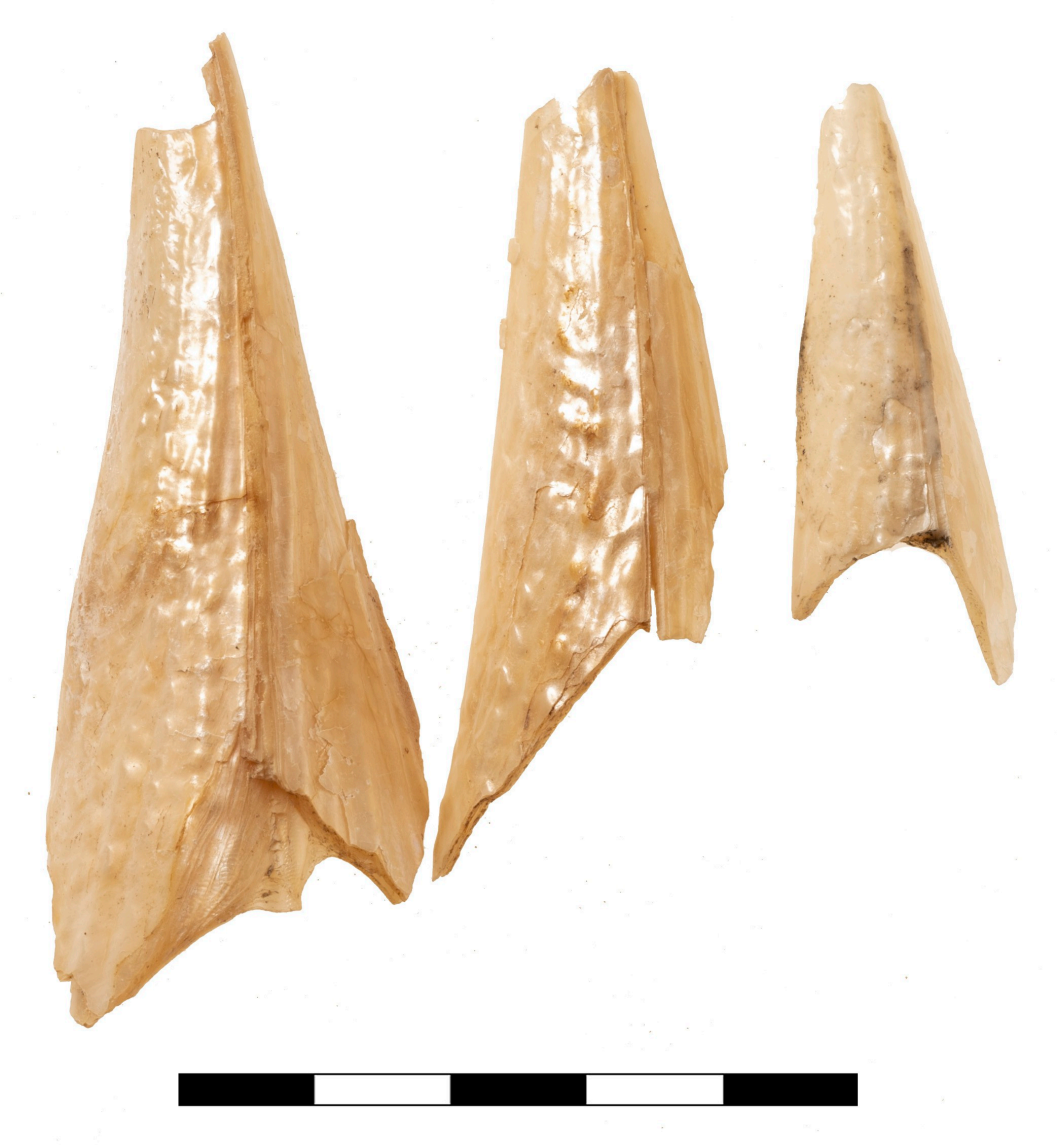
Pen shells from K10. Photo by G. Forstenpointner.

The recovery of marine bivalve remains (Table 6) was almost exclusively processed by dry-sieving. In terms of counted fragments, this sample is largely dominated by the pen shell, *Pinna nobilis* (Linné, 1758). However, due to the extraordinary brittleness of the shell valves, only the calculated MNI gives an impression of the true abundance of the species. As the right and left valves of the pen shell are not distinguishable (Fig 15), the MNI was defined as the half of the determined hinges (n = 109, MNI = 55). Similarly, in terms of MNI, *Arca noae* (Noah’s Ark shell, Linné, 1758) seems to be quite common in the sample (n = 241, MNI = 89 by left valve), while *Spondylus gaederopus* (spiny oyster, Linné, 1758) can be detected somewhat less frequently (n = 138, MNI = 42 by right valve).

**Table 6:** Marine bivalves and other taxa from K10: Dry-sieved samples.

Pteriida	NISP	MNI
*Pinna nobilis* (Linné, 1758)	2036	55
**Pectinida**		
*Spondylus gaederopus* (Linné, 1758)	138	42
*Chlamys varia* (Linné, 1758)	5	3
*Anomia ephippium* (Linné, 1758)	3	3
**Arcida**		
*Arca noae* (Linné, 1758)	241	89
*Arca imbricata* (Bruguière, 1789)	3	2
*Barbatia barbata* (Linné, 1758)	2	2
*Glycymeris glycymeris* (Linné, 1758)	3	1
**Cardiida**		
*Cerastoderma glaucum* (Bruguière, 1789)	8	4
**Venerida**		
*Ruditapes decussatus* (Linné, 1758)	2	1
*Chamelea gallina* (Linné, 1758)	2	2
*Venus verrucosa* (Linné, 1758)	1	1
**Mytilida**		
*Mytilus galloprovincialis* (Lamarck, 1819)	2	1
**total**	**2446**	**205**
**Cephalopoda**		
*Sepia sp*. (Linné, 1758)	4	4
**Echinoidea**		
*Paracentrotus lividus* (Lamarck, 1816)	169	?

The use of the three main species in the bivalve sample must remain open to interpretation, at least in parts: All three taxa are edible, and the Ark shell probably had no other purpose. However, *Pinna* produces a byssus that is the origin of the precious sea silk, a prestigious textile since Aegean Bronze Age [68]. Furthermore, in the archaeological context of possible roof constructions that had covered the younger Early Mycenaean building, clear agglomerations of pen shells have been documented. A possible use of *Pinna* valves instead of roof shingles should not be excluded, even though no comparative data are available. Valves of *Spondylus* provide highly valued raw material for the production of beads or other ornamental objects, although the use of these shells in Late Bronze Age Greece decreased significantly in comparison to Neolithic settlements [69]. We have identified several fragments of *Spondylus* shells that deliberate intentional manipulation, but a mainly alimentary exploitation seems likely. All other identified taxa occur only occasionally and therefore do not imply any particular meaning.

Besides gastropods and bivalves, *Cephalopoda* and *Echinoidea* are also represented, the latter by numerous (n = 169) shell fragments of the purple sea urchin (*Paracentrotus lividus*), the former by four cuttle bones of the genus *Sepia*. These finds also support the impression of an active and multifaceted exploitation of marine food resources.

## Discussion and summary

Frequent deposits of crushed purple snail shells at Bronze Age sites indicate dye production by the local community but not necessarily within the settlement. Shell debris in domestic contexts (especially close to an oven) often represents waste transferred from a dye-production workshop for recycling reason (as temper for pottery, mortar, and plaster, as lime, or simply as drainage layers, cf. [13, 41, 63, 70, 71]). Large-scale workshops or working areas would be expected outside the settlements, ideally located near the shore, due to the high odor nuisance during purple-dye production [41, 62, 63]. These locations would provide sufficient space and easy access to the necessary resources such as fuel, water and raw materials. Additionally, the snails must be kept alive until they are processed [62], which would have required baskets or similar cages in the sea or large containers with seawater, such as the tanks carved into the rocks on the beach of Chryssi Island [72]. The dye processing area, where the snails were crushed and steamed, should not be too far away from these places [63]. Nevertheless, among the earliest examples of known purple-dye production centers in the Aegean region, there is evidence of such activities also within the settlements, most of which are located near or directly on the coast [40, 41, 71–73]. The purple-dye workshop in the settlement of Toumba/Thessaloniki suggests a „small-scale production of purple-dye focusing on community requirements” [40]. No large-scale industry should be expected within these domestic areas.

These observations and assumptions are also consistent with the findings at Kolonna. The archaeological evidence at K10 attests to dye production on site through three indicators: raw material/debris (crushed murex shells), tools/facilities (pounders, grinding stone, waste pit), and finished product (dye pigment). They illustrate several steps of the *chaîne opératoire* associated with the production process [14, 71]. Further significant features in Area K10, such as installations in the form of ditches and basins, or ashes indicating nearby hearths, cannot be directly linked to the production of purple-dye. However, it must be taken into account that K10 does not cover the entire room or area of the presumed workshop; the small, excavated area can only represent a section.

On the one hand, the high degree of fragmentation of the shells from the pit indicates the processing of the whole crushed snail-body, as already mentioned by Pliny the Elder (nat. hist. IX, 126); on the other hand, these conditions largely exclude the alimentary use of the snails as an alternative interpretative approach. In terms of catching methods, the malacological evidence remains ambiguous: the high percentage of small, probably juvenile individuals might indicate the use of baited traps for catching the purple shells, while the remarkable homogeneity of the sample from the pit rather suggests a hand collected catch by diving [62]. No reliable estimate of the scale of purple dye production seems possible, as there is no evidence of the frequency of emptying and refilling of the waste pit. However, the ubiquitous admixture of partly trampled purple shell fragments suggests at least continuous, perhaps professional activities.

The vessels preserved in fragments were obviously used for heating or rather storing the liquid dye. The chemical analysis of the adhering pigment layers by HPLC, together with the malacological record, revealed an overwhelming predominance of *Hexaplex trunculus*, similar to or even higher than in pigments from Wadi Murabba’at [25] or Tel Shiqmona [29]. Possible explanations for this peculiarity of Aeginetan purple-dye industry could be either the higher yield of dye from *Hexaplex trunculus* compared to the other species [39] or unconsciousness of the technological advances offered by the process of “double dyeing”, which requires pigments from both *Hexaplex trunculus* as well as *Bolinus brandaris* (Pliny nat. hist. IX, 134–135; [29]).

Linear B documents attest to the high value of purple-dyed garments in the Late Bronze Age. Tablet X 976 from Knossos attributes the term “royal” to a group of purple-dyers or purple-colored objects [11, 74], in this case associating the process and/or the products with the palace [cf. 75] and, on the other hand, indicating the existence not only of “palatial” but also of “non-palatial” dye-production [11]. Accordingly, the process of dye-production played a significant and multifaceted role in the Mycenaean society and its economy. A common human practice is to facilitate elaborate–and perhaps profitable–processes by means of rituals that are able to avoid the effects of divine anger and the resulting misfortunes [76]. While these rituals very often involve offerings, the recognition of animal sacrifice in the archaeological record depends on finding animal remains of restricted age, sex, species, body part and treatment, as opposed to generalized animal refuse [76]. On the basis of this definition, the interpretation of the holocaustic burnt remains of piglets and lambs within the destruction layers of both Early Mycenaean buildings in Area K10 as offerings to protect the production process of purple-dye does not seem unlikely. Holocaustic burning of piglets in a sacrificial context is known from the Mycenaean sanctuary of Ayios Konstantinos near Methana [77, 78] and from the pre-palatial Megaron B at Eleusis [79], however, a corresponding interpretation of our finds from Area K10 remains hypothetical, as the stratigraphy and archaeological context do not support a clear relationship between the burning of young domesticates and purple-dye production.

At Kolonna, there is no evidence for a specific use or trade of the dye or colored objects, nor can we ascertain any exceptional production for a particular social group. However, the finds from Area K10 not only prove the existence of an intra-urban purple-dye workshop, but also provide new insights into the technological and possibly spiritual background of the process.
